# Working smarter, not harder: silencing *LAZY1* in *Prunus domestica* causes outward, wandering branch orientations with commercial and ornamental applications

**DOI:** 10.1093/hr/uhaf106

**Published:** 2025-04-16

**Authors:** Andrea R Kohler, Courtney A Hollender, Doug Raines, Mark Demuth, Lisa Tang, Macarena Farcuh, Chris Dardick

**Affiliations:** Department of Horticulture, Michigan State University, 1066 Bogue St, East Lansing, MI 48824 USA; Appalachian Fruit Research Station, Agricultural Research Service, United States Department of Agriculture, 2217 Wiltshire Rd, Kearneysville, WV 25430 USA; Department of Horticulture, Michigan State University, 1066 Bogue St, East Lansing, MI 48824 USA; Appalachian Fruit Research Station, Agricultural Research Service, United States Department of Agriculture, 2217 Wiltshire Rd, Kearneysville, WV 25430 USA; Appalachian Fruit Research Station, Agricultural Research Service, United States Department of Agriculture, 2217 Wiltshire Rd, Kearneysville, WV 25430 USA; Appalachian Fruit Research Station, Agricultural Research Service, United States Department of Agriculture, 2217 Wiltshire Rd, Kearneysville, WV 25430 USA; Department of Plant Science and Landscape Architecture, University of Maryland, 4291 Fieldhouse Dr, College Park, MD 20742 USA; Appalachian Fruit Research Station, Agricultural Research Service, United States Department of Agriculture, 2217 Wiltshire Rd, Kearneysville, WV 25430 USA

## Abstract

Controlling branch orientation is a central challenge in tree fruit production, as it impacts light interception, pesticide use, fruit quality, yield, and labor costs. To modify branch orientation, growers use many different management practices, including tying branches to wires or applying growth regulator sprays. However, these practices are often costly and ineffective. In contrast, altering the expression of genes that control branch angles and orientations would permanently optimize tree architecture and reduce management requirements. One gene implicated in branch angle control, *LAZY1*, has potential for such applications as it is a key modulator of upward branch orientations in response to gravity. Here, we describe the phenotypes of transgenic plum (*Prunus domestica*) trees containing an antisense vector to silence *LAZY1*. We found that *LAZY1* silencing significantly increased branch and petiole angles. *LAZY1*-*antisense* lines also displayed ‘wandering’ or weeping branch trajectories. These phenotypes were not associated with decreases in branch strength or stiffness. We evaluated the utility of *LAZY1-antisense* trees for use in two planar orchard systems by training them according to super slender axe and espalier methods. We found that the *LAZY1-antisense* trees had more open canopies and were easier to constrain to the trellis height. This work illustrates the power of manipulating gene expression to optimize plant architecture for specific horticultural applications.

## Introduction

Controlling the orientation of lateral organs is crucial for plant survival in response to changing light environments and competition with other plants. In a horticultural or agricultural context, lateral organ angles impact essential traits such as light interception (through positioning of branches and leaves), drought tolerance (through positioning of lateral roots), and harvest method (through positioning of fruit). Wide branch angles are considered desirable in tree fruit production; they generally reduce bark inclusion, leading to stronger branch unions and an increased ability to support fruit load [1]. Highly branched canopies with wide angles are particularly desirable in some types of high-density, commercial training systems where the focus is on producing a narrow and uniform canopy that can support a high crop load.

Due to the impacts of branch angle on production, growers often expend considerable labor trying to control the position of branches through pruning, tying, and applying growth regulators [2, 3]. However, as the angle of lateral organs is largely determined by genetics, these attempts can be futile. Some species, such as *Prunus domestica* (European plum), exhibit highly vigorous, upright growth habits, making it particularly difficult to train them in high-density planar systems [2]. In contrast to cultural practices, using selective breeding or gene editing to manipulate expression of genes that control lateral organ orientation can provide a permanent solution to branch angle control throughout the lifetime of the plant [2].

One of the primary gene families known to control lateral organ angle is the IGT family, whose members have a conserved (GϕL(A/T)IGT) amino acid motif [4, 5]. The family includes *TAC1* homologs, which promote outward lateral organ orientation in shoots, and *LAZY1* homologs, which promote narrower lateral organ angles [5–18]. Most species have multiple paralogs of *LAZY*, which can be further divided into three subclades: *LAZY1*-like, *DEEPER-ROOTING1*-like (*DRO1*-like), and *LAZY5*-like [5]. These paralogs are partially redundant in controlling shoot and root angles but show distinct expression patterns. *LAZY1* homologs are the most influential in shoots, while the *DRO1*-like genes predominantly regulate lateral root angles.

*LAZY* homolog mutants across plant clades have wider lateral organ angles [5–15]. They also exhibit slowed or absent gravitropic response, sometimes to the point that shoots grow prostrate on the ground—as in *Oryza sativa* (rice) [15], *Zea mays* (maize) [10], and the *Arabidopsis thaliana* (Arabidopsis) *lazy1,2,4* mutant [12]. In some species, *lazy* mutants also have roots that are negatively gravitropic, as in *Medicago truncatula* [11], *Lotus japonicus* [13], and the Arabidopsis *lazy2,3,4* multiple mutant [11]. This suggests that LAZY proteins promote narrowed lateral shoot and root angles in response to gravity. Consistent with this, overexpression of some *LAZY* homologs led to narrower branch and root angles and enhanced gravitropism [16–18]. *LAZY* homologs also are involved in responses to light quantity and quality, likely functioning to integrate light and gravity signaling during the establishment of the default lateral organ angle or ‘set-point angle’ [14, 19, 20].

The function of LAZY proteins in the gravitropic pathway has been extensively explored during the last two decades. Upon gravistimulation, LAZY proteins are phosphorylated by the kinases MKK5 and MPK3 [21]. This promotes the binding of LAZY proteins to TRANSLOCON AT THE OUTER CHLOROPLAST ENVELOPE (TOC) proteins on the outside of amyloplasts [21]. As a result, LAZY proteins become enriched at the plasma membrane at the lower side of gravity-sensing cells (statocytes) as the amyloplasts sediment [21, 22]. Once polarized to the lower side of the cell, LAZY proteins recruit PIN3 auxin efflux proteins, which control polar auxin transport during gravitropism and development [23]. This recruitment is likely through interactions with RCC1-like domain (RLD) proteins [23]. The PIN3 proteins transport auxin laterally in stems and roots to establish gravitropic auxin gradients with greater auxin concentrations on the lower side of gravistimulated roots and shoots. In gravistimulated Arabidopsis *lazy1,2,4* triple mutants, PIN3 mislocalizes to the upper side of statocytes, resulting in an inverted auxin gradient [24].

Here, we present results from a long-term phenotypic study of *P. domestica* (European plum) trees that were transformed with a *LAZY1* silencing construct. This work was initiated >12 years ago for horticultural purposes after identifying *TAC1* as a *Prunus persica* (peach) branch orientation regulator and grouping it with *LAZY1* in the IGT family [4]. Our goal was to test if reduced *LAZY1* expression would result in trees with wider branch angles and more outward orientations. The silencing construct used sequence from peach *LAZY1* (*Prupe.1G222800*) because an annotated genome for *P. domestica,* a hexaploid with a complex lineage, did not exist at that time. A later analysis supports the specificity of our silencing sequence to plum *LAZY1* alleles and not other *LAZY/DRO* plum genes.

Our data includes qualitative and quantitative greenhouse and field data for *LAZY1-antisense* plums on their own roots and on commercial rootstocks. We report on how silencing *LAZY1* influenced plum branch angles, growth trajectories, and branch orientations. In connection, we present the results of a tree-training study that tested the utility of *LAZY1-antisense* trees for planar (2D) commercial orchard systems. Further, we report novel phenotypes related to *LAZY1* silencing impacting wood development and photosynthesis that have important implications for use of such mutants in agricultural applications.

**Figure 1:**
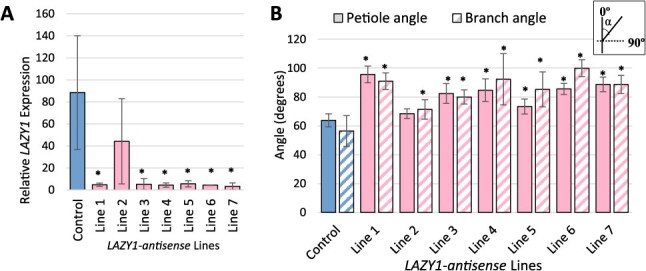
***Prunus domestica* (plum) trees transformed with a *LAZY1-antisense* silencing construct exhibited reduced expression of *LAZY1* along with wider petiole and branch angles**. (A) *LAZY1* gene expression in control and *LAZY1-antisense* lines. Expression was determined by qPCR on at least three biological replicates (trees) per line, each with three technical replicates. Values are in relation to a standard curve of known RNA from control plants. (B) Average branch and petiole angles for *LAZY1-antisense* and control lines. Both petiole and branch angles represent averages from 3 to 10 trees per line. Diagram in the upper right indicates that angles reported are those between the branch or petiole and the apex of the shoot from which it emerged. Bars represent standard deviation and ^*^ indicates a significant difference between the control plants and a *LAZY1-antisense* line (*P* < 0.05) according to a Student’s *t*-test. For A and B, control trees were plum seedlings from same cultivar that did not contain the *LAZY1-antisense* vector.

## Results

### A silencing sequence from peach is specific to *PdoLAZY1* alleles

European plum, like peach, has two independent *LAZY* genes (*LAZY1* and *LAZY2*), and two *DRO* genes (*DRO1* and *DRO2*) (Supplementary Table S1, Supplementary Fig. S1). European plum is a hexaploid species with unknown origin but is thought to be an allopolyploid [25]. Consequently, European plum could have up to six alleles of each *LAZY* homolog. Between four and six alleles were identified for each gene (Supplementary Table S1) [26]. Variation in number of alleles could be due to the collapse of homologous regions in the genome assembly. For *LAZY1*, we identified six distinct alleles, which we named *PdoLAZY1A* through *PdoLAZY1F* (Supplementary Table S1, Supplementary Fig. S1). The amino acid sequences for the first four *LAZY1* alleles (*PdoLAZY1A, PdoLAZY1B, PdoLAZY1C*, and *PdoLAZY1D*) were identical to the peach (*PpeLAZY1*) sequence (Supplementary Fig. S2). The amino acid sequence for the fifth allele (*PdoLAZY1E*) had a single substitution (T48N). The sixth *LAZY1* allele (*PdoLAZY1F*) has a 278-amino acid truncation, as shown in Supplementary Fig. S2. This truncation may be a natural mutation or possibly an assembly error.

We then analyzed the plum *LAZY* and *DRO* sequences to check the specificity of the antisense construct we derived from the *PpeLAZY1* sequence (Supplementary Fig. S3). The 306-nucleotide silencing sequence had >98% identity with each of the five full-length *LAZY1* plum alleles (Supplementary Table S2 and Supplementary Fig. S4). In contrast, there was minimal overlap between the silencing sequence and *LAZY2*, *DRO1*, and *DRO2* alleles. Percent identity between the alleles of all three genes and the silencing sequence ranged from ~14% to 56% (Supplementary Table S2). Additionally, there were no stretches of ≥20 nucleotides that aligned to the *LAZY1* silencing sequence (Supplementary Fig. S4; Supplementary Table S2). Thus, it is likely that our silencing vector is highly specific for *LAZY1*.

### *LAZY1-antisense* trees have wider branch angles and undirected lateral branch growth.

**Figure 2:**
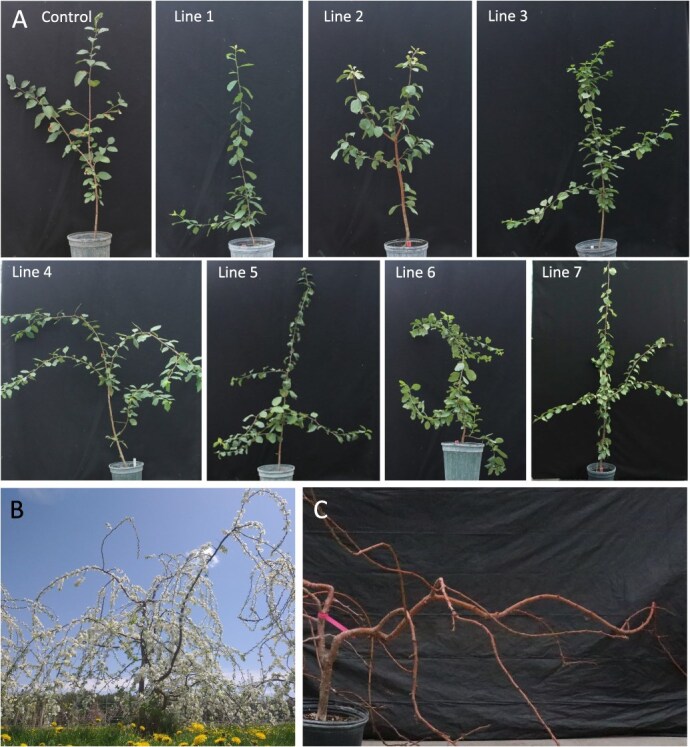
***LAZY1-antisense* plums exhibit altered leaf and branch orientations**. (A) Representative control and *LAZY1-antisense* trees (1–2 years old) from each transgenic line growing in the greenhouse. Each line had a minimum of five trees and a maximum of 13. *LAZY1* expression was not significantly reduced in Line 2. (B) A mature *LAZY1-antisense* Line 6 tree growing in the field. (C) A *LAZY1-antisense* Line 6 tree growing the greenhouse, demonstrating the wandering growth trajectory.

Seven independent plum lines containing the *LAZY1-antisense* vector were generated using agrobacterium-mediated hypocotyl transformation (see methods). *PdoLAZY1* expression was quantified by quantitative polymerase chain reaction (qPCR) using primers with 100% sequence identity to the full-length *PdoLAZY1* alleles, but several mismatches with the nontarget *LAZY/DRO* alleles (Supplementary Fig. S5). In six out of the seven lines (Lines 1, 3, 4, 5, 6, and 7) *LAZY1* expression was significantly reduced (Fig. 1A), and trees from those lines had wider petiole and primary branch (crotch) angles (Fig. 1B). Those lines also had dramatically more horizontal (outward) branch orientations in both greenhouse and field environments (Fig. 2). Line 2 trees did not have reduced *LAZY1* expression, and their branch orientations were upward similar to controls (Figs 1 and 2). As *LAZY1*-*antisense* trees aged, their branches displayed a mix of wandering, arching, weeping, and outward branch phenotypes (Fig. 2B and C).

### Shoots from grafted *LAZY1-antisense* tree buds did not exhibit upward growth

**Figure 3:**
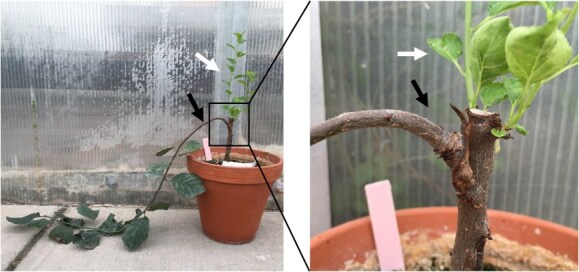
**Shoots from grafted *LAZY1-antisense* buds grew horizontally from standard rootstock.** Representative plum tree with a Line 4 *LAZY1-antisense* branch (black arrow) growing from a bud that was grafted onto a standard ‘Myrobalan’ plum rootstock. Vertical shoots emerging from rootstock buds are indicated by the white arrow.

In preparation for our tree-training field studies, dormant vegetative buds from a representative *LAZY1-antisense* line (Line 4) and a ‘Stanley’ Open Pollinated (OP) plum tree (serving as a control for seedling variation among standard trees) were grafted onto ‘Myrobalan’ plum seedlings in the greenhouse. In some cases, rootstock interactions can influence scion architecture. After bud break, shoots emerging from the *LAZY1-antisense* buds grafted on ‘Myrobalan’ rootstock grew horizontally, as shown in Fig. 3 (black arrows). This indicated that mobile signals from the rootstock could not rescue the branch phenotype. Furthermore, new vegetative shoots growing from the rootstock stem above the graft union were upward orientated. Thus, silencing *LAZY1* in the branch below did not induce a branch phenotype in shoots growing above the graft union (Fig. 3). The inability of *LAZY1-antisense* shoots to exhibit normal upward growth (negative gravitropism) when grafted to a wild-type rootstock indicates that these trees can be used on conventional rootstocks without significantly altering their growth habits.

### *LAZY1*-*antisense* lines show unique characteristics for planar training and revealed an impaired shoot gravitropism phenotype

**Figure 4:**
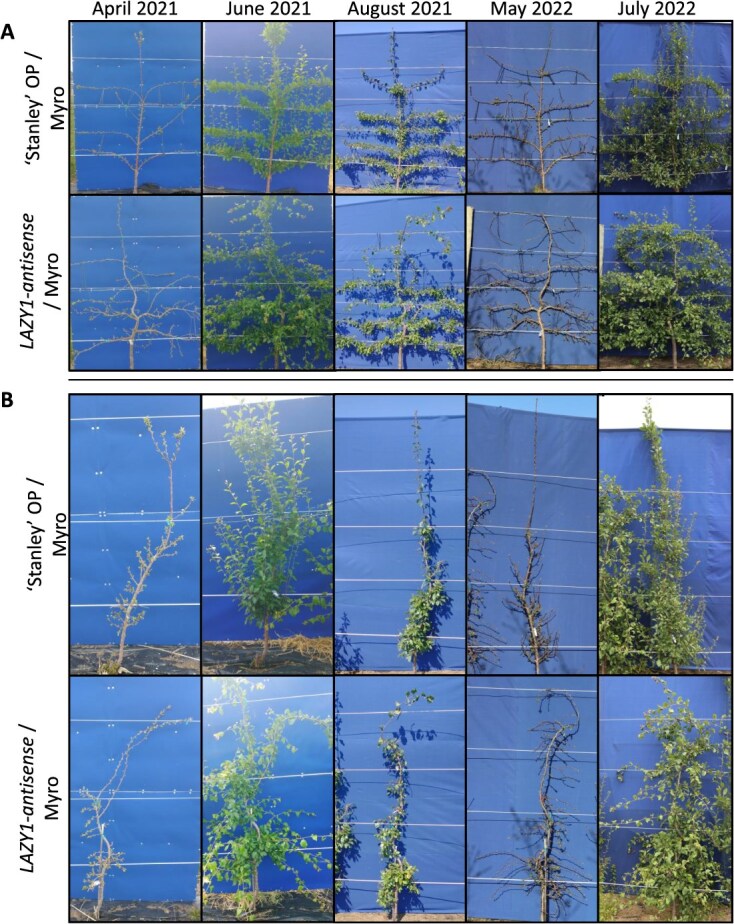
**Representative ‘Stanley’ OP and *LAZY1-antisense* Line 4 on ‘Myrobalan’ rootstock trained into planar systems**. (A) Espalier training. (B) SSA training. Note that ‘Stanley’ OP trees trained as Espalier and SSA grew past the top (fourth) trellis wire by August 2021 while the *LAZY1*-antisense trees grew along it and downwards. ‘Myro’ indicates ‘Myrobalan’ rootstock.

**Figure 5:**
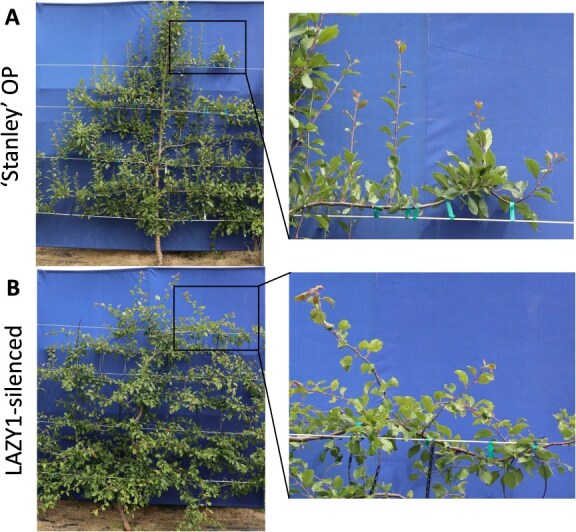
**Lateral branch phenotypes of *LAZY1-*antisense trees**. (A) When ‘Stanley’ OP branches were tied horizontally for espalier training, they continued trying to reorient upwards and buds broke from the top of the branch. (B) *LAZY1-antisense* branch tips did not reorient upwards, and buds broke from all sides of the branch.

To test performance of *LAZY1-antisense* plum trees in planar training systems, the *LAZY1*-antisense Line 4 trees on ‘Myrobalan’ rootstock were trained in the horizontal palmette espalier system used in fruit trees for hundreds of years [27], as well as the super slender axe (SSA) system designed for sweet cherry (Figs 4 and 5) [28]. Trees were trained and observed over the course of 5 years. Tree trunks (which originated as a shoot from a grafted bud) were forced to maintain a vertical orientation by tying them to support stakes and trellis wire at the time of planting and regularly throughout each season.

The trunks from the ‘Stanley’ OP controls consistently grew straight up (Fig. 4). These trees generally sent up a single leader with limited lateral branches, which rapidly grew above the trellis (Fig. 4). Heading this leader provoked a strong flush of growth at the top of the tree, shading the lower tree and draining vigor away from where it is desired lower in the tree (Fig. 4). In contrast, the trunks of the *LAZY1-antisense* trees wandered side-to-side and/or arched downward at sites where they were tied to trellis wire or their supportive stake (Fig. 4). Because *LAZY1*-*antisense* trunks had to be staked upright for vertical growth, they did not grow significantly taller than the trellis (Fig. 4). Further, heading the main trunk did not provoke a flush of vegetative growth at the top of the tree (Fig. 4).

The actively growing branch tips of lateral branches from control trees trained to maintain a 90-degree orientation in the espalier study were consistently oriented upward, exhibiting classic negative gravitropism (Fig. 5A). Secondary shoots growing from lateral buds on control tree branches also exhibited upward growth (Fig. 5A). However, *LAZY1-antisense* tree branch shoot tips and secondary shoots did not show any clear gravitropic response or consistent directional growth (Fig. 5B). In addition to growing in every direction, secondary shoots from the horizontal *LAZY1-antisense* branches emerged from buds on every side of the tied branches, and not just buds on the upper side, as they did in control trees (Fig. 5B).

### Branches from *LAZY1-antisense* trees do not have reduced stiffness or strength, but their wood has altered material properties.

**Figure 6:**
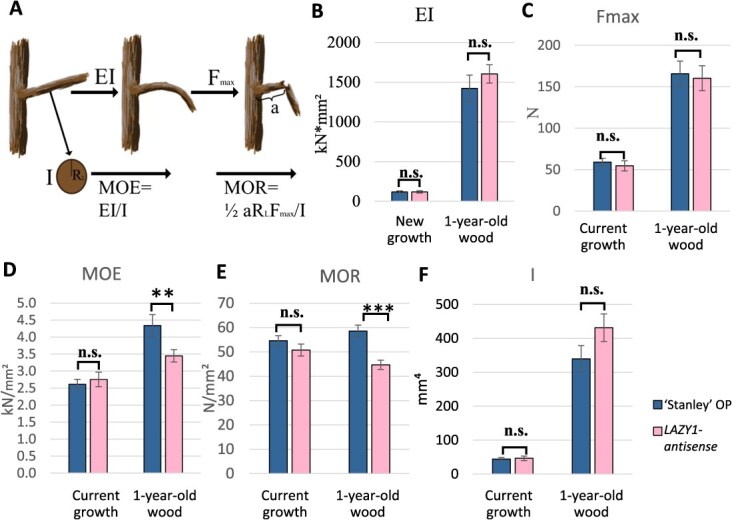
**Biomechanical properties of current year growth and 1-year-old branches from *LAZY1-antisense* trees**. (A) Diagram illustrating how biomechanical properties relate to bending force and failure. (B–F) Biomechanical properties of *LAZY1-antisense* Line 4 branches compared to ‘Stanley’ OP control branches: (B) flexural stiffness (EI); (C) maximum force (Fmax); (D) modulus of elasticity (MOE); (E) modulus of rupture (MOR); and (F) area moment of inertia. Bars represent standard error. Branches taken from four trees per genotype. *n* = 30 per genotype for new growth, *n* = 13 for ‘Stanley’ OP first year, and *n* = 14 for *LAZY1-antisense* first year. Comparisons between genotypes done with pairwise *t*-tests. ^*^ indicates significantly different at α = 0.10, ^**^ indicates significant at α = 0.05, ^***^ indicates significant at α = 0.01, n.s. indicates not significant at α = 0.10.

Xylem differentiation and development are associated with polar auxin transport [29], and LAZY proteins influence auxin transport in response to gravity [8]. Therefore, we investigated the biomechanical properties of wood from our *LAZY1-antisense* plum trees. To do this, we performed materials testing experiments on comparable new growth and 1-year-old branches from the ‘Stanley’ OP control and *LAZY1-antisense* Line 4 trees on the ‘Myrobalan’ rootstock (Fig. 6A). We found that compared to controls, the *LAZY1-antisense* tree branches did not have any alterations in their ability to resist bending (flexural stiffness; EI) (Fig. 6A and B). Nor was there a change in the maximum force that their branches could resist (F_max_) (Fig. 6A and C). Thus, the wandering phenotype of the branches is not due to floppiness, or an inability to hold themselves upright. Despite branch stiffness (EI) and strength (F_max_) remaining constant, the material properties of the wood from *LAZY1-antisense* branches were different than wood from ‘Stanley’ OP branches. The 1-year-old *LAZY1-antisense* wood had a lower modulus of elasticity (MOE) indicating that it was more flexible (Fig. 5D). It also had a lower modulus of rupture (MOR), which indicates weakness, or a greater likelihood that it would break from a given force (Fig. 6E). However, the lower MOE and MOR did not reduce the overall stiffness or strength of the *LAZY1-antisense* branches because they had increased area moment of inertia (I), which is a measure of cross-sectional area, accounting for how the area is distributed relative to the force; Fig. 6F).

### *LAZY1-antisense* trees exhibit leaf chlorosis and reduced net photosynthesis.

**Figure 7:**
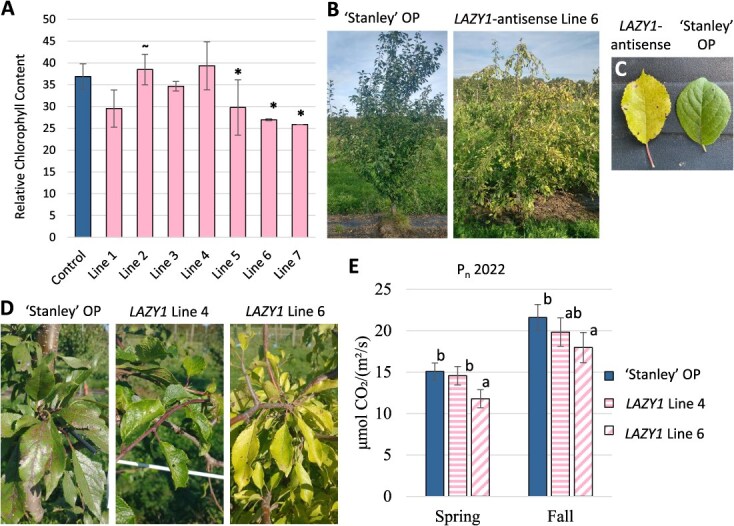
***LAZY1-antisense* trees had reductions in chlorophyll content and net photosynthesis** (A) Chlorophyll content of *LAZY1-antisense* lines in Kearneysville, WV. The ˜ serves as a reminder that Line 2 did not have significant reduction in *LAZY1* expression. ^*^ Indicates *P* < 0.05. (B) A ‘Stanley’ OP control and a *LAZY1-antisense* Line 6 tree growing in Clarksville, MI. (C) Leaf chlorosis comparison for Stanley and *LAZY1-antisense* Line 6 in Kearneysville, WV. (D) Leaf photos taken fall 2021 in Clarksville, MI. (E) Net photosynthesis in spring versus fall for 2022 growing season in Clarksville, MI. Means within the same time point with the same letter are not significantly different at α = 0.05. Thirty or more measurements were taken for each Genotype^*^Timepoint combination.

Toward the end of each growing season, we observed leaf chlorosis in field-grown *LAZY1-antisense* lines growing in both Kearneysville, WV, and Clarksville, MI (Fig. 7A–D). The phenotype was somewhat variable. The extent and timing of the chlorosis depended on the line, year, and location, suggesting a Genotype-by-Environment interaction. For example, chlorosis was regularly observed in Lines 1, 5, 6, and 7, but generally not in Line 4 trees (Fig. 7D). To quantify the chlorosis, relative chlorophyll content was measured for all lines in 2021 (Fig. 7A). We also investigated if the chlorotic tendency impacted photosynthesis. In both spring and fall 2022, net photosynthesis for Line 4 and Line 6 trees growing in Michigan was measured. Consistent with the chlorophyll measurements, net photosynthesis was reduced in Line 6, but not in Line 4 (Fig. 7E). Interestingly, Line 6 trees had decreased photosynthesis at both time points, despite their leaves not showing chlorosis at the spring time point.

### *LAZY1-antisense* plums produced normal fruit

After several seasons in the field, the *LAZY1-antisense* plum trees flowered and fruited in both West Virginia and Michigan. Due to the change in canopy shape and potential influence of the observed chlorosis, we investigated potential impacts on fruit quality. All lines produced flowers and fruit that were visually comparable to control trees (Supplementary Fig. S6). A preliminary fruit quality assessment was done for fruit from five lines growing in West Virginia in 2024 by determining their soluble solid content (SSC). The *LAZY1-antisense* lines exhibited a slight decrease in SSC (Supplementary Fig. S6), however the values were still within the normal range for plum seedlings [30].

## Discussion

This long-term study began with plum tree transformations initiated in 2012, included two field studies, one planted in West Virginia starting in 2015, and another planted in Michigan in 2018, and ended with preliminary fruit evaluations in 2024. Our results demonstrated that reducing *LAZY1* expression can effectively alter branch angle in a tree fruit crop. We observed *LAZY1-antisense* plums at all stages of development and under diverse conditions, bringing the observations closer to real-world application. *LAZY1-antisense* lines exhibited increased branch angles and orientations from the seedling stage through maturity. This phenotype was stable when grafted onto standard rootstock, grown in locations with different climates, and after various pruning regimes.

These *LAZY1-antisense* lines displayed multiple traits that are beneficial for commercial production. The wider branch angles resulted in more open canopies that could increase light penetration, improving flower bud development and fruit quality [31]. An unexpected benefit of *LAZY1-antisense* trees came from their more bush-like growth habits (Fig. 2B). The main trunk did not grow upright, there was no clear leader, and the trees grew more horizontally than vertically. This unexpectedly solved one of the central problems in planar systems, which is keeping the trees at or below the height of the trellis without stimulating excessive vegetative vigor by heading the tree [27]. The agravitropic growth noticeably facilitated planar training by limiting tree height to the highest point at which it was tied, decreasing bursts of localized vegetative vigor, and increasing uniformity of the canopy. Control of vigor is particularly important in European plum, as extremely vigorous shoots generally do not produce floral buds [3]. Crucially, *LAZY1-antisense* lines did not have reduced branch strength or stiffness, indicating they maintain the ability to support heavy crop loads.

These *LAZY1-antisense* lines also exhibited phenotypes that may limit their applicability for commercial production but could prove useful for ornamental applications. The undirected growth of *LAZY1-antisense* branches presents training challenges. For espalier with control trees, horizontal branches can be trained between wires via counteracting negative gravitropism (upward growth) by tying the branches to the wire below. As *LAZY1-antisense* trees do not have strong gravitropic responses, it was difficult to train branches between wires; they needed to be tied to the wires below and above to maintain their trajectory (Figs 4 and 5). From a training perspective, the problem can be solved by placing a trellis wire at each interval where horizontal growth is desired and tying the branch directly to it. The wandering habit also causes idiosyncratic changes in direction of the main trunk, which made it difficult to create a uniform canopy.

When allowed to develop freely, the wandering branch phenotype produces very attractive trees for ornamental use (Fig. 2B). Wandering branches, as in corkscrew willows and Young’s weeping birch, are prized for adding winter interest to landscaping. Interestingly, this phenotype has not previously been reported in *LAZY1* knockdowns, even in woody species such as apple [14]. However, this phenotype may not be limited to plum. A correlative study suggested that the weeping trait in Young’s weeping birch (*Betula pendula* ‘Youngii’) is the result of a premature stop codon in a *LAZY1* homolog [32]. While this study does not describe a wandering phenotype, it is clearly visible in photographs of this cultivar available from arboretums and nurseries. This twisting phenotype may be related to circumnutation, especially as previous studies have shown that rice *lazy1* mutants have decreased circumnutation [33]. However, it is unclear why these windings are ‘frozen in time’ and remain visible in the woody branches.

The role of auxin transport in wood development and the changes we observed in the biomechanical properties of first-year branches from *LAZY1-antisense* trees may point to a role for *LAZY1* in wood development. Alternatively, because the changes in biomechanical properties and diameter are consistent with what is observed in flexure wood or tension wood, these changes may be a secondary effect of the unique *LAZY1-antisense* branch orientation, with branches forming reaction wood and/or flexure wood in response to their horizontal trajectory.

Multiple *LAZY1-antisense* lines unexpectedly showed reduced chlorophyll content (Fig. 7) and leaf chlorosis. Chlorosis in at least one of these lines was associated with a decrease in net photosynthesis. While undesirable for production systems, their unique color may be a desirable trait for ornamental use. The variance of the phenotype among lines and seasons suggests that the trait may not have 100% penetrance, which may assist in selecting the desired phenotype. Leaf chlorosis has not been previously reported for *lazy1* mutants. This may be because most studies on *lazy1* mutants were short-term experiments using annual species grown in growth chambers. In plum, the chlorotic phenotype only became apparent midway through the growing season in the high light intensity of the greenhouse and field. Given recent revelations about the ability of LAZY1 protein to associate with the TOC1 complex found in amyloplasts and other plastid membranes, as well as its role in light response/signaling [21, 22], the leaf chlorosis may indicate a role for LAZY1 in the response of photosynthesis to light-related signaling, the absence of which results in chlorosis. This hypothesis is strengthened by the observation that photosynthesis is reduced in the spring in chlorotic Line 6, prior to any observable leaf chlorosis.

In the ongoing quest to produce tree fruit more efficiently, advances that improve tree structure and fruit yield while decreasing labor inputs will be crucial to success. Due to the substantial amounts of labor required to physically constrain and control tree canopies over the lifespan of the orchard, modifying the genetics controlling canopy structure will be a key part of long-term solutions. Here we have demonstrated that manipulation of *LAZY1* expression can permanently alter canopy structure, providing traits beneficial to planar training systems. While some of the pleiotropic phenotypes we observed were deleterious to commercial production, this work provides a strong foundation for further investigation of genetic manipulation of branch angle in tree fruit.

## Materials and methods

### *LAZY* gene identification and phylogeny

*LAZY* genes in *P. domestica* were identified using BLASTp of the previously identified peach LAZY proteins [5] against predicted proteins from the *P. domestica* draft genome v1.0 (Fig. 1 and Supplementary Table S1) [26]. Due to a highly unusual exon–intron structure, *LAZY* genes are often misannotated [34]. This structure is conserved across species, with each gene containing five exons, with the first exon including just two codons (often an ‘ATGAAG’) and the last exon containing only ~20 bases [34]. To identify and correct errors in annotation, the DNA sequences of plum genes identified as *LAZY* homologs were aligned to the homologous peach and Arabidopsis sequences. Using that alignment, the plum and peach genes were reannotated with particular attention to identifying a gene model that fit the conserved gene structure. Following reannotation, the protein sequences were predicted, and the resulting protein sequences were used for subsequent alignments.

Alignments and phylogenetic trees were produced in CLC Genomics Workbench v22.0. Alignments were performed using a gap extension cost of 1.0, and a gap open cost of 10.0, the alignment set to ‘Very accurate’. The protein phylogenetic tree was generated using the Neighbor Joining method with 100 bootstrap replicates and Jukes–Cantor as the protein distance measure. For Supplementary Table S2, alignments were performed using Clustal Omega.

### Cloning

To silence *LAZY1* in *P. domestica* (European plum), hypocotyls extracted from ‘Stanley’ plum seeds were transformed with a silencing construct containing a 306-nucleotide sequence from the peach *LAZY1* gene (*PpeLAZY1;* peach genome version 1.0 ID ppa007017, now named Prupe.1G222800 in genome version 2) in the reverse orientation behind a 35S promoter. A peach sequence was used because an annotated plum genome was unavailable at the time. After the publication of the European plum genome [26], *LAZY* and *DEEPER ROOTING* (*DRO*) homologs were identified by protein BLAST searches using peach *LAZY* and *DRO* sequences (see Supplementary Table S1; Supplementary Figs S1 and S2). The gene fragment was amplified using primers PpLazy-1 1F (5’AAGCCAAACTGTGGCACAAAGC) and PpLazy-1 2R (5’AGCTGCCAGGACTTTCTCCAA T), cloned into pENTR-D TOPO (Invitrogen, Carlsbad, CA), and from there into the pHellsgate 8.0 vector [35] using LR Clonase (Invitrogen, Carlsbad, CA). While the intended construct would have had two copies of the 306-bp fragment as a hairpin, the construct used for transformation was later discovered to only have a single insert in the reverse orientation behind the 35S promoter within pHellsgate 8.0, creating an antisense vector rather than an RNAi construct.

### Plum transformation

The pHellsgate 8.0 vector with the *PpeLAZY1* fragment was transformed into *Agrobacterium tumefaciens* GV3101 and then engineered into European plum (*P. domestica* L) according to Petri *et al*. [36]. Briefly, cold (4°C) stored seeds from OP ‘Stanley’ plum trees were cracked to remove their endocarp, surface sterilized with 15% bleach for 15 min and washed with sterile water three times. Hypocotyl slices were excised under a laminar flow hood using a stereomicroscope. After a 20-min incubation with the *Agrobacterium* suspension, the transformed hypocotyl sections were cultured in cocultivation medium for 3 days. The hypocotyl sections were then transferred to antibiotic (80 mg/l kanamycin) selection medium. Resulting kanamycin-resistant (transgenic) shoots were multiplied in plum shoot multiplication medium, rooted on rooting media, and then acclimated in a growth chamber before being transplanted into 15- to 23-cm pots in a greenhouse. Each line contains trees that originated from the same cluster of callus on a hypocotyl slice, and between 5 and 13 trees were generated per line.

### Plant material

Greenhouse-grown *LAZY1-antisense* and control lines were used for quantifying gene expression and for branch and petiole angle analysis. Trees were 1–2 years old when gene expression was quantified and petiole angles were measured, and 2–3 years when branch angles were measured. The controls for *LAZY1* expression were transformed plum seedlings from OP plum cultivar ‘Stanley’ (‘Stanley’ OP) that did not contain the *LAZY1-antisense* vector. These plums instead contained the pSUC/PSUL vector, which does not impact branch angle [37].

*LAZY1-antisense* plum trees and ‘Stanley’ OP controls were planted at the USDA-ARS Appalachian Fruit Research Station (AFRS) research field in Kearneysville, WV, and at the Michigan State University Clarksville Research Center (CRC) near Clarksville, MI. The trees at AFRS were planted at ~2.4-m spacing in October 2014. The *LAZY1-antisense* plums were planted in two blocks of three trees. Control plums were planted between groupings of *LAZY1-antisense* trees. The trees for training studies at CRC were generated in 2017 and 2018 from budwood from *LAZY1-antisense* Line 4 and ‘Stanley’ OP control taken from AFRS and grafted onto ‘Myrobalan’ rootstock. After a year and a half of greenhouse growth, the grafted trees (0.3–0.5 m in height) were planted in September 2018. For horizontal palmette espalier training, trees were planted at ~2.4-m spacing, and for SSA training they were spaced at ~0.9 m. These trees were trained to trellis with ~46-cm spacing between wires. The CRC planting also contains the *LAZY1-antisense* Line 6 trees on their own roots.

### RNA extraction and gene expression analysis

The E.Z.N.A SQ Total RNA Kit (Omega Bio-tek, Inc., USA) was used to extract total RNA from frozen leaf tissue samples for *LAZY1* expression analysis. RNA was treated with DNase I to remove contaminating genomic DNA. To determine gene expression levels, qPCR reactions were carried out using the gene-specific primers PpLAZY-qPCR-5F (5’ ATGCTTTATGCTTCTTCTCG) and PpLAZY-qPCR-5R (5’ TTGCTCAGCAGATGAGGT; Supplementary Fig. S3) and the Super Script III Platinum SYBR Green One-Step qRT-PCR Kit with ROX (Invitrogen Corp., USA). The qPCR was run using an ABI 7900DNA Sequence detector (Applied Biosystems) using the following protocol: cDNA synthesis step was performed at 50°C for 5 min, followed by a PCR starting with 95°C for 5 min, then 40 cycles of 95°C for 15 s and 60°C for 30 s, followed by 40°C for 1 min. The qPCR was performed on RNA from three to four independent biological replicates (trees) for each transgenic line, as well as on five control trees. Each biological replicate had three technical replicates for the reactions. A standard curve generated from serial dilutions of RNA from a tree that did not have the *LAZY1* vector was run at the same time to determine relative expression values.

### Petiole and branch angle measurements

Petiole angles for up to 10 leaves growing from the trunk of young (<1-year-old) trees that had not yet initiated lateral shoots were measured in 2013 using a protractor. For the control and Lines 3 through 7, five trees were measured. Ten trees were measured for Line 1, and three trees for Line 2. Crotch (branch) angles for six branches from 3 to 10 trees per genotype growing at AFRS were measured in 2021 using a protractor. Angles represent the angle between the branch and the shoot from which it emerged. When the branch angles were measured, the trees were ~9 years old and had been growing in the field for 6 years.

For both petiole and branch angle statistical analyses, the average angle per tree was considered a biological replicate, and standard deviations are based on those averages. A *P*-value <0.05 from Student’s *t*-test was used to determine significant differences between the *LAZY1-antisense* lines and control trees.

### Fruit-soluble solids measurements

Between 15 August and 9 September 2024, 6–15 fruit were harvested from six Stanley trees and three to six trees for each *LAZY1-antisense* line (except Line 1, for which only one tree with fruit was available). A digital handheld refractometer (PAL-1, Atago, Tokyo, Japan) was used to measure Soluble Solid Content, and firmness was measured using a Lutron FR-5120 digital penetrometer with an 8-mm tip. The average brix for each line was modeled in R 4.4.1 using the ‘lme4’ package with the equation Brix = μ + Line + Tree + Firmness + e, where μ is the grand mean, ‘Line’ was the effect of the line, ‘Tree’ was the effect of the individual tree, ‘Firmness’ was a linear variable, and ‘e’ was the residual error. This model was gated using analysis of variance (ANOVA) and marginal means for lines were estimated using the ‘emmeans’ package. *t*-tests were performed against Stanley using the ‘multcomp’ package.

### Biomechanics measurements

Clonal trees were generated from budwood from a single OP ‘Stanley’ (‘Stanley’ OP) tree and a single *LAZY1-antisense* Line 4 tree grafted onto ‘Myrobalan’ rootstock, grown at CRC and trained as horizontal palmette espalier. Horizontally oriented new growth and first-year wood (branches initiated the previous season) were collected 9 July 2023. Four to five branches per tree were collected from four trees per genotype for new growth, for a total of 20 ‘Stanley’ OP and 19 *LAZY1-antisense* samples. Three to five branches per tree were collected from three trees per genotype for first-year samples, for a total of 13 ‘Stanley’ OP and 14 *LAZY1-antisense* samples.

An Instron Universal Testing Machine (Model 4202, Instron Corporation, Canton, MA) with a 500 N load cell was used to conduct four-point bending tests. The span of the outer supports was set to 90 mm, while the two posts of the actuator were set to 30 mm. Leaves and lateral branches were removed from a section of wood ~10 cm long, and the section was oriented so that the upper side of the branch was positioned down on the Instron, so that force was exerted in the same direction as gravitropic force acted on the branch in its original position. The force versus displacement curve was measured until a maximum force was reached.

The equation EI = (F/V)(a^2^/12)(3L–4a) was used to quantify Flexural stiffness (EI), with (F/V) representing the slope of the linear section of the force/displacement curve, ‘a’ representing the post-to-load distance, and L represented the total length of the span. Flexural stiffness was then used to calculate the MOE, using the equation MOE = EI/I, where I is the area moment of inertia. For a branch with an elliptical cross section, I = (π/4) (R_P_)(R_L_^3^), where R_L_ is the radius in the direction of loading and R_P_ is the radius perpendicular to loading. The MOR was determined using the equation MOR = (1/2 Fmax)(a)(R_L_)/I, where Fmax was the maximum force withstood by the branch. A Student’s *t*-test was performed to test for statistically significant differences between the genotypes using the T.TEST function in Excel, assuming a homoscedastic two-tailed distribution.

### Chlorophyll and photosynthesis measurements

Chlorophyll measurements were taken from field-grown trees at the AFRS with a SPAD 502 Chlorophyll Meter (Konica Minolta, Inc., Tokyo, Japan). For 2021, six leaves were measured per tree for 3–10 trees per genotype, on 25 August.

Photosynthetic parameters were measured in late spring (9 and 23 June 2022) and early fall (5 October 2022) using a CI-340 infrared gas analyzer (CID Bio-Science, Camas, WA). The measurements were taken with an open system in differential mode, with 6.25 cm^2^ of leaf area in the chamber, and a flow rate of 0.3 l/min. Measurements were taken from trees at CRC, including control trees (‘Stanley’ OP on ‘Myrobalan’ rootstock and on own root), *LAZY1-antisense* Line 4 on ‘Myrobalan’, and *LAZY1-antisense* Line 6 trees on their own roots. At each time point, three to five measurements were taken per leaf, on three to five leaves per tree, with two to six trees per genotype.

Statistical analysis was performed in R v4.3.1. To control for the environmental variability of the field conditions, net photosynthesis (Pn) for all genotypes was modeled as a response to photosynthetically active radiation (PAR), the concentration of carbon dioxide at intake (CO2in), air temperature (Tair), air pressure (Pressure), and water vapor pressure (H2Oin) at intake. Externally studentized residuals were used to identify influential outliers, and observations with a studentized residual >|2| were dropped (10 in total). Studentized residuals and the criterion Cooks Distance for point>3(mean Cooks distance) was used to identify outliers. Outliers were then manually checked. Outliers for which there were two or more outliers per leaf were retained as reflecting true biological variability, while single outliers were omitted. The model was then rechecked without outliers. Variance inflation factor (VIF) was used to check for collinearity of the environmental variables. Added variable plots were used to check for linearity and the contribution of each additional variable. Variables with the least contribution or the highest VIF were sequentially dropped until VIF < 3 for all variables, and all variables had a clear contribution to the model. Finally, variables for genotype and for accounting for the repeated measures were added to the model.

The resultant model for photosynthesis was Pn = μ + Genotype + Timepoint + Genotype*Timepoint + (1|Genotype:Tree) + (1|Genotype:Tree:Leaf) + PAR+ CO2 + Tair + Pressure + error where μ indicates the grand mean, ‘Genotype’ is a variable for the control or RNAi line, ‘Timepoint’ accounts for the effects spring versus fall, ‘Genotype^*^Timepoint’ accounts for the interaction between genotype and time point, (1|Genotype:Tree) controls for the effects of tree, which is a random variable nested within genotype, (1|Genotype:Tree:Leaf) controls for leaf-to-leaf variation, and is nested in tree, and ‘PAR’, ‘CO2in’, and ‘Tair’ are continuous variables controlling for their respective environmental factor. This model was gated with ANOVA, and normality and equal variances of the residuals were checked. Since interaction between genotype and time point was significant, all pairwise comparisons were performed for genotype slicing by time point using *t*-tests at α = 0.05.

## Supplementary Material

Web_Material_uhaf106

## Data Availability

The data underlying this article are available in the article and in its online supplementary material.
